# All Iron Battery 3.0

**DOI:** 10.1016/j.ohx.2025.e00629

**Published:** 2025-02-03

**Authors:** Dipak Koirala, Surendra K. Gautam, I. Francis Cheng, Peter B. Allen

**Affiliations:** a Department of Chemistry, University of Idaho, 875 Perimeter Dr, MS 2343, Moscow, ID 83844, USA; b Department of Chemistry, Tri-Chandra Multiple Campus, Tribhuvan University, Kathmandu, Nepal

**Keywords:** Battery, Renewable electricity, Grid scale storage, Redox mediators, Open source

## Abstract

Battery storage technology can address a key limitation to renewable energy. Renewable electricity generation (solar and wind) is intermittent. An inexpensive energy storage device with excellent rechargeability and safety is critical for grid applications and for the global transition to renewable energy. In this work, we introduce an energy storage secondary battery based on an aqueous all-iron chemistry with redox mediators. The cell employs commodity chemicals methyl viologen and 2,2′-azino-bis(3-ethylbenzothiazoline-6-sulfonic acid) (ABTS) at the anode and cathode, respectively. The result is a highly rechargeable, low-cost energy storage system with a good price-performance ratio compared to commercial rechargeable batteries that is stable for 100+ cycles with 84 % capacity retention. The cell has a volumetric capacity of 9.6 Ah/L (energy of 11.52 wh/L) and power density of 72 Watts/m^2^.

## Graphical abstract

**Figure:**
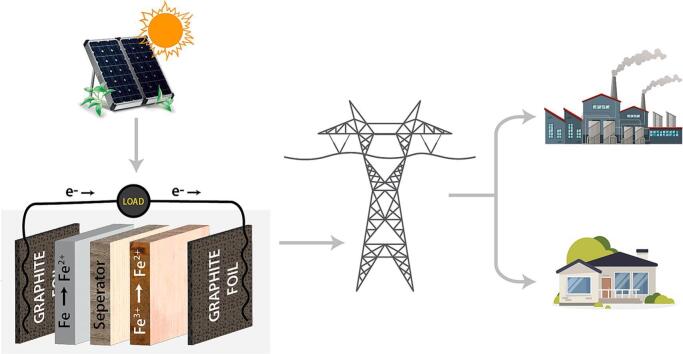

Specifications table

**Table:** 

Hardware name	*All Iron Battery 3.0*
Subject area	● Engineering and materials science
Hardware type	● Other: battery
Closest commercial analog	*Lead acid gel battery*
Open source license	*CC-BY-4.0*
Cost of hardware	*$4.59*
Source file repository	*https://doi.org/10.17605/OSF.IO/D3N2J*

## 1 Hardware in context

Net zero carbon emission goals require a large-scale expansion of renewable energy. However, this requires methods to match asynchronous demand with the intermittent supply of solar and wind power. Power buffering can be accomplished with electrochemical batteries. Ideally, these systems should be inexpensive, use environmentally green materials, have durability greater than 10 years, and have sufficient energy density [1], [2]. Furthermore, systems based on aqueous electrolytes should be emphasized over organic solvents.

A variety of aqueous battery chemistries have been examined for power buffering. These include redox flow batteries, lithium-ion and sodium-ion batteries [3]. The present state of stationary power buffering systems may benefit from further improvements to cost-per-kwh, system longevity, lower maintenance requirements and safety. It is important to note that power and energy densities are secondary considerations for stationary storage. Aqueous all-iron batteries have several characteristics that fulfill requirements for stationary battery systems. Iron is abundant, inexpensive, relatively nontoxic, and environmentally friendly. Iron also has several oxidation states (0, +2, +3 and +4) that enable it to be used as both the positive and negative battery electrodes. Although all-iron batteries have been reported, these systems may suffer from one or more of the following, high costs, limited lifetime (<100 cycles), and/or low power density (0.0001–0.01 mW/cm^2^) [4], [5], [6].

The benefit of an open-source battery is not just lower cost energy storage, it is also accessibility. While not competitive with the power density of lithium ion, the performance is now in a useful range for practical applications. An open source battery with useful performance characteristics means that other open energy hardware (e.g., wind turbines, solar cells, micro-hydro or portable sensors and monitoring equipment) could be built outside of the normal supply chains and advanced manufacturing associated with modern high performance batteries.

## 2 Hardware description

The All-Iron Battery:

• Can be integrated into any device that uses DC current
• Supplies approximately 1 V under load and is rechargeable at 1.25 V
• Construction is compatible with oxygen and moisture

We previously demonstrated an All-Iron Battery (AIB)1.0 and 2.0[7], [8]. The previous designs incorporated aqueous paste electrodes with high concentrations of conductive carbon at near-neutral pH. The electrodes were designed based on the following proposed reactions.

Negative electrode: Fe(0) *(s)* + 2OH^–^ *(aq)* → Fe(OH)_2_ *(s)* + 2e- (1)

Positive Electrode: 2Fe(OH)_3_ *(s)* + 2e^-^ → 2Fe(OH)_2_ *(s)* + 2OH^–^ *(aq)* (2)

Cell Reaction (1.2 V): Fe(0) *(s)* + 2Fe(OH)_3_ *(s)* → 3Fe(OH)_2_ *(s)* (3)

The All-Iron Battery (AIB) 2.0 featured low-cost electrode materials in safe 2 M K_2_SO_4_ electrolyte. It was stable for 1000 shallow cycles (5 % of capacity)[8]. However, the power density was too low (0.002 mW/cm^2^) for most applications. Based on a review of the literature, this may be attributed to the slow kinetics of electron transfer between the iron species within both the positive and negative electrode pastes[6], [9], [10]. In this contribution for the AIB 3.0 we improve performance by the incorporation of redox shuttles that allow charge transfer between the iron species within the positive and negative pastes. These redox mediators have standard reduction potential similar to the active materials. Methyl viologen (MV^0/2+^) was used in the negative paste electrode. Diammonium 2,2′-azino-bis(3-ethylbenzothiazoline-6-sulfonate) (ABTS^0/2-^) was used in the positive paste electrode. These are commodity chemicals (a herbicide and common analytical reagent, respectively) and are available at large scale for low cost.

MV^2+^ *(aq)* + 2e^-^ → MV *(aq)* E^0^ = -0.45 V (4)

ABTS *(aq)* + 2e^-^ → ABTS^2-^ *(aq)* E^0^ = +0.75 V (5)

**Fig. 1:**
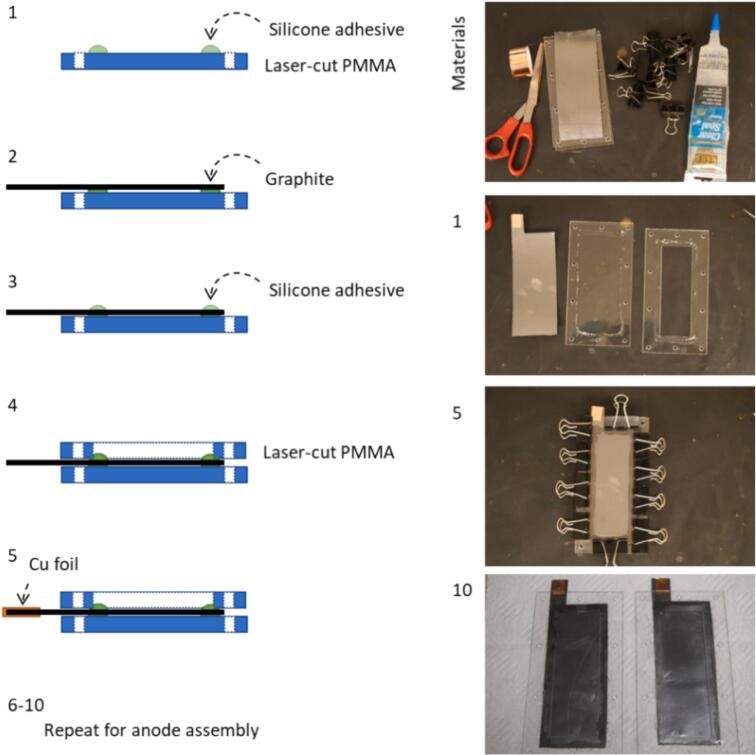
Overview of all-iron battery. (A) Schematic showing the battery chemistry during discharge with ferric iron and metallic iron being converted to ferrous iron at the positive and negative electrode, respectively. (B) Photograph of the all-iron battery during operation lighting two LEDs simultaneously (2 LED of 0.03 Watts were glowing for 1 h of tested time).

The similarity of the reduction potentials ensures the ability to shuttle charge between iron species with different oxidation states within the paste electrodes. The scheme is shown in Fig. 1. In this iteration (AIB 3.0), additional steps were to minimize the hydrogen evolution reaction (Reaction 6). This parasitic reaction degrades battery performance and is a safety hazard [11].

Hydrogen Evolution: 4H_2_O *(l)* + 4e^-^ → 2H_2_ *(g)* + 4OH^-^ *(aq)* E (pH7) = -0.414 V (6)

## 3 Design files summary

**Table:** 

**Design file name**	**File type**	**Open source license**	**Location of the file**
*2 ml-cell.dxf*	CAD	Public Domain (CC0 1.0 Universal)	https://doi.org/10.17605/OSF.IO/D3N2J

**Fig. 2:**
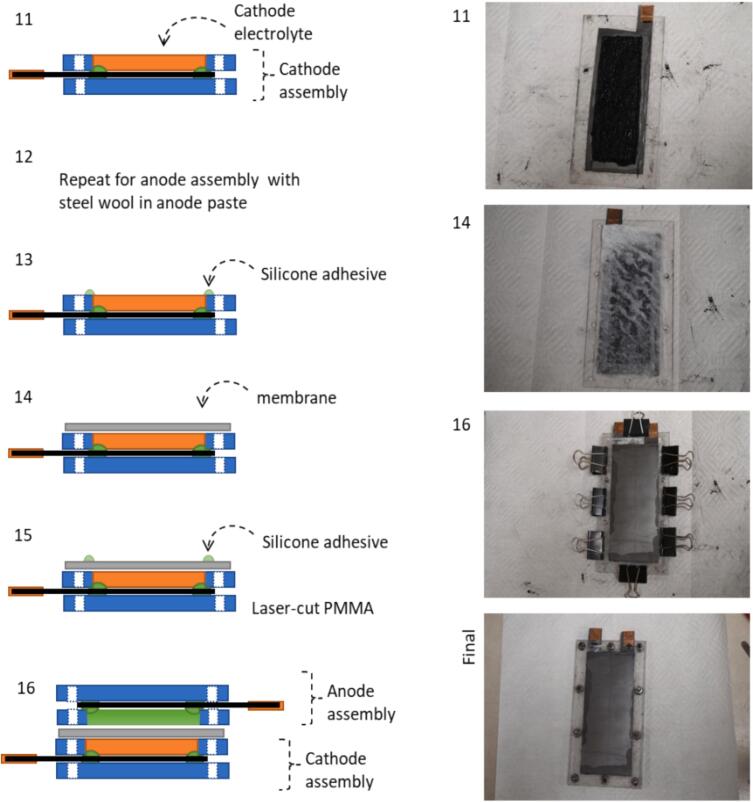
Illustration of 2 ml cell assembly for using the laser-cut acrylic parts specified in *2 ml-cell.dxf*.

2 ml-cell.dxf: Pattern for cutting a 2 ml half-cell housing from 1.6 mm thick acrylic (PMMA) sheet. Two copies should be cut for each cell. Instructions for assembly of the cell from these components are included in section 3 of Supplemental Information. In brief, the design and its application are shown in schematic in Fig. 2.

## 4 Bill of materials summary

The following table indicates retail sources and prices to reproduce the battery chemistry at small scale. Wholesale prices are lower and are considered later in Performance and Validation.

**Table:** 

**Designator**	**Component**	**Number**	**Cost per unit −$**	**Total cost −S/xxx unit**	**Source of materials**	**Material type**
*Iron Compound*	*Iron(II) chloride*	∼2 g	0.53	66.65/250 g	Alfa Aesar	Inorganic
Iron Metal	Iron Powder	0.2 g	0.05	59.40/250 g	Fisher Chemical	Metal
Base	Potassium Hydroxide	1.75 g	0.2	56.60/500 g	EMD Millipore	Inorganic
Electrolyte	Potassium chloride	0.30 g	0.03	111/kg	Fisher Chemical	Inorganic
ABTS	2,2′ −Azino-bis-(3-ethylbenzothiazoline-6-sulfonic acid), diammonium salt	5.5 mg	0.33	120/2g	Sigma-Aldrich	Organic
MV	Methyl viologen dichloride hydrate	2.75 mg	0.11	191.00/5g	Fisher Chemical	Organic
	Hydroxylamine Hydrochloride	6.95 mg	0.04	125/25 g	Sigma-Aldrich	Organic
EG	Ethylene Glycol	0.40 mL	0.01	79.10/gal	VWR Chemicals	Organic
Current Collector	Graphite Sheet	13 cm^2^	0.01	22/m^2^	Mineral Seal Corporation	Composite/Semiconductor
Separator	Fumasep FAS-50	6.25 cm^2^	1.1	80/600 cm^2^	Fuel Cell Store	Composite
Adhesive	Clear Seal Sealant	∼0.5 g	0.02	6.95/5.5 oz	Local hardware store	Composite
Housing	Acrylic Sheet	64 cm^2^	0.25	35/m^2^	Local hardware store	Plastic
Ketjen Black EC-600JD [KB]	EC-600JD	0.2 g	0.3	69.50/50 g	Nouryon, Amsterdam	Inorganic
	Copper Tape	∼2 cm^2^	0.01	20/m^2^	Walmart	Metal
	Nuts and Bolts	8 pc each	1.6	0.10/pc	Local hardware store	Plastic/metal/alloy
Total Cost		4.59				

## 5 Build instructions

The build instructions are adapted from the All-Iron Battery 2.0[8] with several modifications. Detailed build instructions including chemicals, instrumentation, and test procedures are included in the Supplemental Information. Key differences from the previous version include an updated cell housing, updated active materials, and the use of a commercial separator membrane.

Briefly, pastes of the active materials for the positive and negative electrodes were prepared separately. These pastes included conductive carbon, precipitated iron salts, electrolyte, and mediators. The pastes were packed into plastic housings with graphite current collectors. A separator membrane was then soaked in electrolyte and placed between the housings and the assembly was held together with machine screws and sealed with silicone sealant.

The cell housing was PMMA (acrylic sheet) with total internal volume of 2 mL (cathode and anode cavity each measured 2.5 × 2.5 × 0.16 cm^3^). The graphite sheet was used as the current collector and Fumasep FAS-50 anion exchange material was used as a separator. The cell was then left for ∼24 h to come to equilibrium before any characterization and performance measurements were made. The active material has been optimized as compared to the previous iteration as explained in Section 7, Validation and Characterization.

## 6 Operation instructions

The AIB 3.0 operates as a standard rechargeable battery, and supplies DC current. Once constructed, the battery can be connected to electronic equipment by wire leads. Multiple batteries can be connected in series or parallel depending on the needs of the application. Charging can be carried out at constant, regulated voltage (1.2 v per cell in series) or using a programmed charge controller.

Safety considerations during battery construction: Hands and any exposed skin should be washed thoroughly after handling. Proper personal protection equipment (PPE) including eye protection and gloves should be worn during handling. Dust/fume/gas/ mist/vapors/spray should be avoided; do not eat, drink or smoke when using the battery.

• Potassium hydroxide (KOH) is used during construction and is corrosive. Can cause skin burns and eye damage.
• Ferrous chloride causes serious eye damage and skin burns. Avoid contact with skin.
• Methyl Viologen is a toxic herbicide and can cause serious eye and skin irritation. May cause respiratory irritation. It should be used in a well-ventilated environment with PPE.
• Hydroxylamine hydrochloride is corrosive and toxic. Causes skin and eye irritation. May cause an allergic skin reaction.
• Ethylene glycol is harmful if swallowed and should be kept away from pets and livestock.

## 7 Validation and characterization

### 7.1 Suppression of the hydrogen evolution reaction (HER)

The HER (Reaction 6) must be minimized for safety and battery life. The HER reaction can be driven by the dissolution of Fe(0), Reaction 7. A variety of measures were examined for their ability to control the HER. These are discussed below.

Fe^2+^ *(s)* + 2e^-^ ⇋ Fe^0^ *(s)* E^0^ = -0.44 V (7)

#### 7.1.1 Effect of cation

**Fig. 3:**
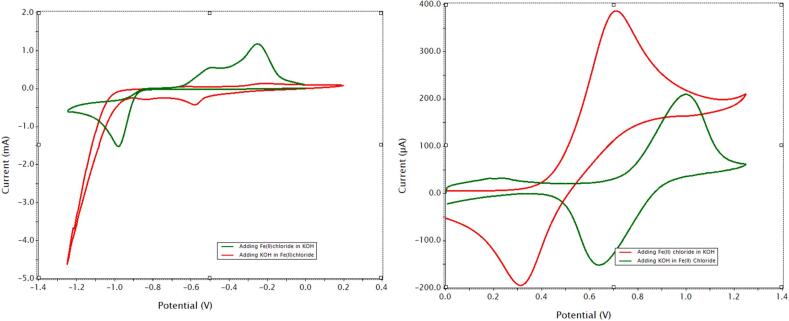
Optimization of the electrolyte composition for suppression of the HER. (A) Linear sweep voltammograms (LSV) of various 2.0 M electrolyte cation chloride salts. Li^+^ and K^+^ have the highest HER overpotentials. (B) LSV graphs from potassium salts of various anions with Cl^-^ having the most cathodic HER onset, (C) Graph of the 1 mA/cm^2^ HER onset potentials for KCl concentrations between 1 M to saturation. (D) Graph of the 1 mA/cm^2^ HER onset potentials for increasing ethylene glycol (EG) composition. The maximum EG concentration of 20 % v/v is dictated by the solubility of KCl.

Electrolyte solutions containing 2 M salts of monovalent, divalent, trivalent metal cations and ammonium ions were examined for their effects on the HER. In this study all anions were chloride. Linear sweep voltammograms (LSV) at 50 mV/s are shown in Fig. 3A. We evaluated electrolytes based on the onset potential at a current density of 1 mA/cm^2^. Of the seven electrolytes tested, Li^+^ and K^+^ were best at HER suppression with onset potentials beyond −0.9 V. In general, monovalent metal ions performed better than divalent and trivalent ones. The range for onset potentials for the various cations was 400 mV. The K^+^ was selected for the AIB 3.0 as 2 M KCl conductivity was greater than the corresponding Li^+^ salt. This is expected based on limiting molar ionic conductivities[12]. We also noted that the cell performed better when iron chloride was added to the potassium hydroxide rather than the other way around (see Supplemental Information Section 5.1).

#### 7.1.2 Addition of methyl viologen does not affect the HER

One of the possible downsides to MV is additional hydrogen evolution as reported in literature [13]. To evaluate this, chronoamperometry was performed at –1.2 V vs. Ag/AgCl for 1 h. No significant hydrogen evolution was observed, and the pH of the electrolyte remained 7.82 before and after the experiment.

#### 7.1.3 Anion effects on the HER

Common anions were examined for their effects on the HER. These were SO_4_^2-^, NO_3_^–^ and Cl^-^. The LSV plot in Fig. 3B shows that chloride has the most negative HER onset potential of −1.1 V at 1 mA/cm^2^. The effect of KCl concentration on the HER is shown in Fig. 3C.

#### 7.1.4 Suppression of HER with aqueous ethylene glycol

Aqueous ethylene glycol (EG) electrolyte was examined in the AIB 3.0 as it is known to suppress the HER [14], [15], [16]. Furthermore, ethylene glycol will also improve the operational temperature range of the aqueous electrolyte with lower freezing and higher boiling points and increases the low temperature conductivity of electrolyte [17]. Although EG is an organic solvent it is widely used with common routes for waste disposal and management. Fig. 3D illustrates the suppression of the HER with ethylene glycol (EG) composition. The 20 % (v/v) EG composition was selected for the AIB 3.0 because higher concentrations reduce KCl solubility. The addition of EG also results in higher current density as measured by cyclic voltammetry (see Supplemental Information section 5.4). The optimized electrolyte for AIB 3.0 is 2 M KCl in 20/80 v/v EG/H_2_O. This increases the HER onset to −1.23 V vs Ag/AgCl which is an improvement compared to −1.08 V in the AIB 2.0 system.

### 7.2 Redox mediators

**Fig. 4:**
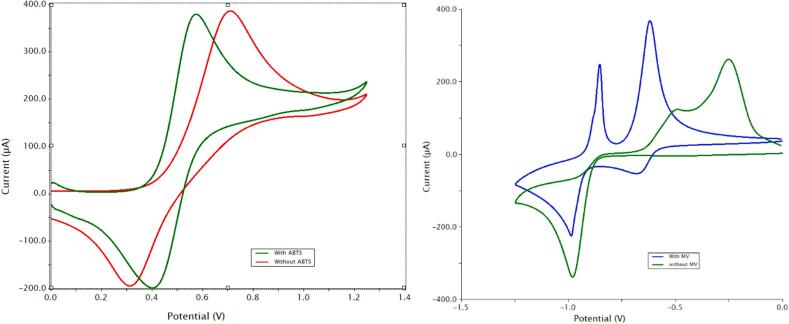
Cyclic voltammetry (50 mV/s) studies in 2 M KCl, 20 % ethylene glycol (v/v) with a 3 mm diameter glassy carbon disk electrode. Top, 10 mM redox mediators, the MV^1+^ state undergoes an adsorption on the glassy carbon surface. Center, potential window. Bottom, positive and negative electrode pastes. The vertical dashed lines demonstrate that the half-wave potentials for the negative and positive pastes’ iron oxidation states match those of the redox mediators.

Soluble redox species can increase power density by efficiently transferring electrons to and from the insoluble iron. The redox mediators (MV^0/2+^ and ABTS^0/2-^) were discussed in the introduction, see Fig. 1 and Equations 4 and 5. This concept was based on studies in the literature demonstrating redox mediators in redox flow batteries [18]. Fig. 4 shows the cyclic voltammograms (50 mV/s) of freely dissolved 10 mM MV^2+/0^ and ABTS^0/2-^ couples (top) and the paste materials without the redox mediators (bottom). The potential window (middle) was tested under N_2_ purge. The cyclic voltammogram demonstrates that the solvent system was stable under the prevailing potentials of the AIB 3.0. The MV^1+^ adsorption peak is typical for many types of electrode surfaces [19]. It is not known how it may affect the redox relay characteristics of the negative electrode (Equation 1), however the MV^0/2+^ relay system does enhance power density as discussed below. The CV half-wave potentials of the redox relays (Fig. 4, dashed vertical lines) align with the ones associated with the negative and positive paste (Fig. 4, bottom) indicating that they are a good match for the scheme outlined in Fig. 1. We also note that the redox mediators improve the kinetics of the electroactive iron species as measured by cyclic voltammetry (see Supplemental Information Section 5.2).

### 7.3 AIB 3.0 longevity at cycling at 0.1C

The previous iteration (AIB 2.0) was shallow-cycled for 1000 cycles at 5 % depth-of-discharge with 8 % loss in capacity. AIB 3.0 battery showed negligible degradation with 400 shallow cycles. We took the stability of AIB 3.0 to be improved over AIB2.0 based on this comparison during shallow cycling. We decided to test its degradation in more realistic circumstances. We applied deep-cycling conditions to determine its limits.

**Fig. 5:**
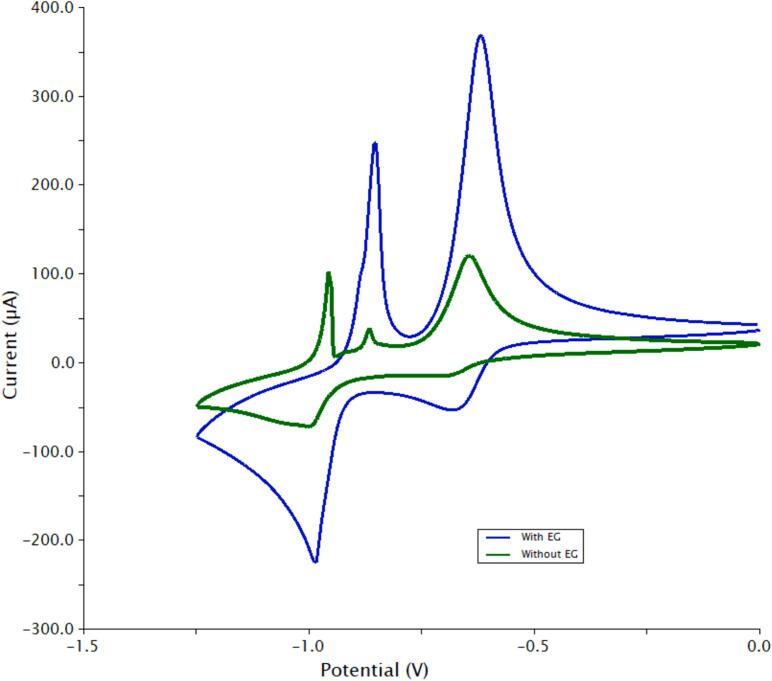
Electrochemical Performance of AIB 3.0 Cell. (Left) Cell capacity vs potential at certain cycles of a cell for a 2 mL cell. The discharge was performed at the constant current of 0.32 mA/cm^2^ (0.1C). (Right) Capacity retention and coulombic efficiency of a cell. The current is 0.32 mA/cm^2^ × 6.25 cm^2^ area electrode (2.0 mA). After 100 cycles the cell has 85.4 % of the initial capacity with columbic efficiency greater than 90 %.

An AIB 3.0 of 2 mL volume was constructed and was fully charged at 1.25 V using a DC power supply. It was then discharged until the potential drops to 0.5 V at a constant current density of 0.32 mA/cm^2^. The charge–discharge current density (0.32 mA/cm^2^) was chosen at 0.1C (10 h) rate, which is the typical discharge rate for stationary applications [20], [21]. The initial cell capacity (19.2 mAh), Fig. 5A, for the 1st cycle was determined from this full discharge. The cell was then charged-discharged at current density of 0.32 mA/cm^2^ at 80 % capacity for 100 cycles, simulating 1 year of use. At the 1st, 25th, 50th and 100th cycles, the cell was fully charged to 1.25 V and fully discharged to 0.75 V to determine the capacity. The other cycles were performed between 1.15 to 0.85 V. This is based on standard industrial protocol[22]. The change in capacity (Fig. 5A) went from 19.2 to 16.2 mAh from the 1st to 100th cycle. Battery storage during this cycling protocol goes from 9.6 to 8.1 mAh per ml. This battery retained 84 % of its initial capacity after 100 cycles with 94 % columbic efficiency (Fig. 5B). This is similar to other stationary systems [23], [24], [25], [26], [27], [28], [29], [30], [31].

The battery capacity during discharge is always less than its capacity during charge. This is due to the combination of many factors such as electrolyte decomposition, change in pH, change in crystal structure of active material, including the self-discharge of the battery. Columbic efficiency (CE%) is the measure of this capacity between each cycle and is given by:

$$CE\left(\%\right)=Q_{disch\arg\arg\;e\;}/Q_{ch\arg\arg\;e\;}\times 100\%$$

where, Q_discharge_ = discharging current × discharging time and Q_charge_ = charging current × charging time. 2 mL AIB 3.0 at 100 cycles was fully charged in 16.48 h and fully discharged in 15.52 h at a charge/discharge current of 2 mA. The calculation is shown in Fig. 5B. Change in columbic efficiency at various cycles is shown in Fig. 5B.

Crossover of the mediators through the membrane will degrade performance. The possibility of this unwanted feature was examined by cyclic voltammetry on an electrochemical cell divided by the Fumasep FAS-50 anion exchange membrane in the 2 M KCl 20 % (v/v) EG AIB 3.0 electrolyte. The initial concentrations were 10 mM MV^2+^ in the left chamber and 10 mM ABTS^2-^ in the right. A constant potential of 1.25 V was applied across the cell with 6.25 cm^2^ graphite electrodes for 24 h. This simulates the AIB 3.0 conditions. After the 24 h period CV was performed on catholyte to evaluate MV crossover and vice versa. We found no crossover through the membrane by the cyclic Voltammetry experiments. After 24 h no ABTS was detected in the left chamber and no MV in the right chamber. This indicates no crossover at the detection limit of CV, which is estimated to be 10 nM.

### 7.4 Energy and power density

**Fig. 6:**
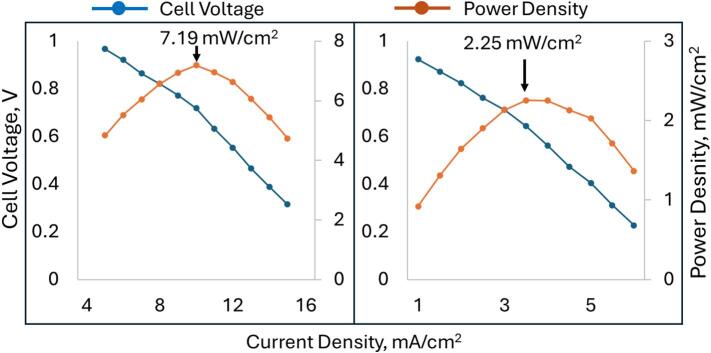
Cell Voltage, Current Density and Power density at 80% State of Charge (SOC). Left is with redox mediators. Right is without redox mediators.

Maximum cell power was determined in the study featured in Fig. 6 as a function of current density. At 80 % state of charge, the battery could deliver 23 W/L or 72 W/m^2^ (for a cell of 2 ml and 6.25 cm^2^). The power curve for the cell is shown in Fig. 6. The new maximal volumetric power density is a significant improvement over the previous iteration of the all-iron cell (which was 92 times smaller at 250 mW/L). While this is still low by the standards of lithium-ion cells, it is nonetheless a promising candidate for stationary energy storage.

**Fig. 7:**
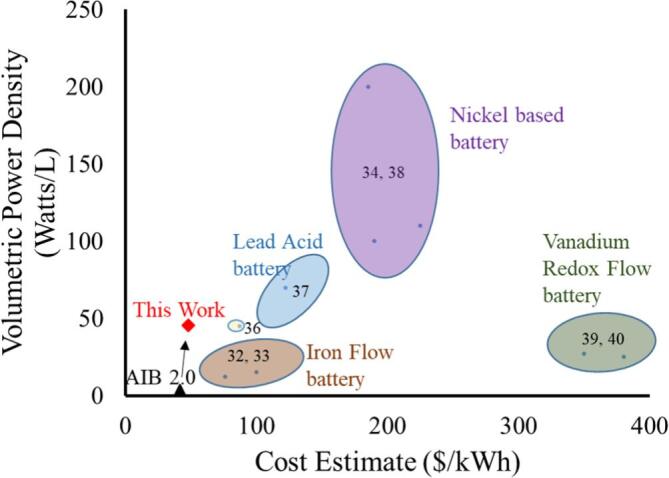
Wholesale Cost vs Power density comparison of various aqueous battery technologies.

Fig. 7 shows a scatter plot of energy density and energy capacity per dollar [8], [32], [33], [34], [35], [36], [37], [38], [39], [40]. The extremely low cost and high abundance of iron, low-cost separator, and industrially available mediators make for very high capacity per dollar. The graph only includes aqueous batteries, which are typically lower in energy density than batteries with organic electrolytes. Typical, commercial Li-ion batteries with organic electrolytes have ∼300 w h/kg, while cutting edge chemistries can reach 700 w h/kg. Aqueous Li-ion batteries have been demonstrated with typical energy densities at 50 w h/kg [41] while record-setting work from Yang et al (2019) demonstrated an aqueous lithium ion system with 460 wH/kg [42].

### 7.5 Final performance metrics

A lower amortized cost of storage is the key driving feature of this work: a stable, low-cost, highly rechargeable energy storage system. Wholesale prices for solar electricity (under the most favorable conditions) have reached as low as $0.02 per kilowatt-hour. If low-cost solar is combined with low-cost battery solutions (such as the work presented here), it could enable dispatchable, baseload renewable electricity. As a rough approximation, an amortized cost of storage at $0.09 per kilowatt-hour would enable solar-storage at cost-competitive rates in favorable regions. Our current prototype has an estimated lifetime of ∼250 cycles (based on extrapolating capacity decay to 60 %. Using standard calculations of battery lifetime based on coulombic efficiency, we estimate approximately 1000+ cycles of operation[43]. Our costs are ∼$50 per kilowatt-hour, giving a range of $0.05-$0.20 per kilowatt hour amortized storage cost. Given the early stage of the battery, we feel that this is a very promising direction for further development.

The electrochemical performance is increased in all respects over previous iterations. It is increased in terms of power density and stability and slightly increased in energy density. The all-iron battery has a low energy and power density compared to commercial batteries, but its performance is suited to stationary applications. We have measured a volumetric energy of 9.6 Ah/L (energy of 11.52 wh/L) and power density of 72 Watts/m^2^. A 10 MWh battery would take up 868 m^3^ or approximately 13 shipping containers. It could deliver ∼20 MW peak power, acting as a “battery backup” to a 2 MW solar farm to allow such a farm to operate on a nearly 24/7 basis. This would have no moving parts and could be installed in a remote location near generation or transfer infrastructure.

A similar system based on conventional lithium-ion batteries would be approximately ten times smaller, but more concentrated energy storage presents new hazards, especially fire risk and toxic electrolytes. AIB 3.0 battery technology has safety advantages over Li-ion and Na-ion batteries. Physical damage to conventional batteries, over-charging and over-discharging, high vibration environments, and even poor manufacturing quality can lead to internal short circuits. In many types of batteries, this can cause thermal runaway. This can result in fires that are hard to extinguish, as has been noted in news reports of electric vehicle and home backup battery fires. Other battery chemistries can outgas toxic chlorine or explosive hydrogen.

The battery chemistry presented here is inherently safer. This battery is resistant to these problems due to its high thermal mass and low intrinsic flammability. Li-Ion batteries have an extremely narrow operating temperature range of between 15 and 45 °C. Our system has a much wider operation window of −10 °C to 60 °C. Additionally all-iron chemistry presents significant environmental benefits. It uses aqueous chemistries and abundant, inexpensive, and non-toxic materials that are relatively benign (i.e., compared to heavy metals and organic electrolytes). This makes the recycling and disposal of an all-iron battery potentially feasible at lower cost. The environmental contamination from disposal is minimal. Besides safety and cost, the production of this battery technology is less energy intensive and clean room manufacturing is not required. These all lead to an overall reduction in the carbon footprint during manufacturing.

Further research could expand the list of iron salts and iron complexes that can be used for cell construction. Further subjects also include a better seal on the battery cell container and compensation for any long-term water losses (e.g. recombination catalysts). We also hope that additives might further increase power density by speeding charge transfer through the electrolyte.

## 8 Ethics statements

This work does not use human or animal subjects.

## CRediT authorship contribution statement

**Dipak Koirala:** Writing – original draft, Visualization, Software, Methodology, Investigation, Conceptualization. **Surendra K. Gautam:** Investigation, Conceptualization. **I. Francis Cheng:** Writing – review & editing, Validation, Supervision, Resources, Methodology. **Peter B. Allen:** Conceptualization, Methodology, Validation, Writing – review & editing, Supervision, Project administration.

## Declaration of competing interest

The authors declare that they have no known competing financial interests or personal relationships that could have appeared to influence the work reported in this paper.
