# Supplementary material for: All Iron Battery 3.0
Source: HardwareX. 2025 Feb 3;21:e00629. doi: 10.1016/j.ohx.2025.e00629 (PMC11869601; doi:10.1016/j.ohx.2025.e00629)
Supplement: Supplementary Data 1 [file mmc1.docx]

**Supplemental Information: All-Iron Battery 3.0**

Dipak Koirala^a^, Surendra K. Gautam^b^, I. Francis Cheng^a^, Peter B. Allen^a*^

^a^ Department of Chemistry, University of Idaho, 875 Perimeter Dr, MS 2343, Moscow, ID 83844, USA

^b^ Department of Chemistry, Tri-Chandra Multiple Campus, Tribhuvan University, Kathmandu, Nepal

^*^Corresponding author, peter@peterallenlab.com

**1. Laser-cut Cell Housing**

Cell housing was adapted from AIB 2.0[1]. Acrylic sheets [Housing] were laser cut per CAD drawings (available at repository https://osf.io/8kwcf/). Acrylic plastic of desired thickness can be purchased from a hardware store or online as Plexiglass brand or similar. As an alternative, the sheets can be ordered pre-cut from a commercial laser cut prototyping service. 1.6 mm thick acrylic (poly methyl methacrylate, PMMA) was used. Any plastic or paper backing should be removed from the laser cut sheets before use.

The included design will generate a cell with an enclosed volume of 2.0 ml. This cell will produce 1.2 V and hold 19.2 mAh. The dimensions can be altered to enclose a larger or smaller volume. Thicker and thinner plastic may change the performance characteristics such as maximal current per unit volume.

# 2. Materials and Methods

# 2.1. Chemicals

Iron (II) chloride tetrahydrate (98%), lithium chloride (99%), and graphite rod (6mm diameter, 99% metals basis) were obtained from Alfa-Aesar (Ward Hill, MA). Iron (III) chloride anhydrous (98%) was obtained from Beantown Chemical (Hudson, NH). Potassium hydroxide (ACS reagent grade), aluminum chloride (ACS reagent grade), magnesium chloride hexahydrate (ACS reagent grade), sodium chloride (ACS reagent grade), and ammonium chloride (ACS reagent grade) were obtained from EMD Millipore (Burlington, MA). Potassium chloride (99.9%), iron powder (99%), calcium chloride dihydrate (ACS reagent grade), potassium sulfate (99%), potassium nitrate (99%), and methyl viologen dichloride hydrate (MV, 98% purity) were obtained from Fisher Chemicals (Hampton, NH). 2,2′ -Azino-bis-(3-ethylbenzothiazoline-6-sulfonic acid), diammonium salt (ABTS, 98% purity) and hydroxylamine hydrochloride (99%) were obtained from Sigma-Aldrich (St. Louis, MO). Ethylene glycol (99%) was obtained from VWR Chemicals (Atlanta, GA). Graphite sheet (0.030” thickness) was obtained from Mineral Seal Corporation (Tucson, AZ). Fumasep FAS-50 anion exchange membrane was purchased from Fuel Cell Store, Texas, USA. Clear seal sealant (Liquid Nails) and 1.6 mm acrylic sheet (generic) were purchased at a local hardware store. Vegetable oil (soybean, WinCo brand) was obtained at a local grocery store to prepare GUITAR coated carbon black accordingly to previously published procedure[2]. Ketjen black (KB) was denoted EC-600JD (Nouryon, Amsterdam) and ball-milled for 24 h before use. All other chemicals were used as obtained without further purification. Ultrapure water of 18.2 MΩcm^-1^ was used to prepare solutions, which was obtained by purifying house deionized water through the Synergy® water purification system (Millipore Sigma, France).

Chemical Safety hazards and PPE:

- Gloves and eye protection should be worn while handling all chemicals.
- Potassium hydroxide (KOH) is used during construction and is corrosive. Can cause skin burns and eye damage.
- Ferrous chloride causes serious eye damage and skin burns. Avoid contact with skin.
- Ethylene glycol is harmful if swallowed and should be kept away from pets and livestock.

# 2.2. Instruments

GUTAR coating on KB microparticles was performed on Thermo Scientific tube furnace (MA, USA) following a previously reported chemical vapor deposition method.^12^ Electrochemical measurements were performed using a Pine Research Wave Driver 100 potentiostat (Durham, NC) using experimental parameters as noted below.

# 2.3. Cyclic Voltammetry (CV) Experiments

CV experiments were conducted in a three-electrode undivided cell with a graphite rod counter electrode, Ag/AgCl (3.5 M KCl, 0.205V vs SHE) reference electrode, and glassy carbon (3 mm diameter) working electrode. All electrolytes were prepared under nitrogen atmosphere. Cyclic voltammetry experiments were performed at a scan rate of 50 mV/s. Hydrogen evolution reaction (HER) experiments employed a 4 mm diameter iron rod working electrode in various electrolytes.

Electrochemistry Safety hazards and PPE:

- Gloves and eye protection should be worn while handling all chemicals.
- Electrochemical experiments where hydrogen may be evolved should be conducted in a location with good ventilation

# 2.4. Positive Electrode Paste Preparation

Oxidized iron (II/III) was obtained by slowly adding 1M FeCl_2_ [Iron Compound] to 3M KOH [KOH] solution under continuous stirring in an ice bath. The solution was further stirred at room temperature for 1 hour. It was filtered, washed with DI water, dried in an oven at 80 °C and stored for further use. AIB 3.0 electrolyte solution should be prepared as follows: 2 M KCl in 20/80 v/v EG/H_2_O. Cathode paste was prepared in batches using 2 g of iron compound solid, 0.1 g of KB in 5 mL AIB 3.0 electrolyte solution at pH 7.5-8.0 containing 10 mM ABTS.

# 2.5. Negative Electrode Paste Preparation

Oxidized iron (II) was obtained by slowly adding 1M FeCl_2_ [Iron compound] to 3M KOH [Base] solution kept in ice bath under continuous stirring. The solution was continuously purged with nitrogen to prevent oxidation. Solid hydroxylamine hydrochloride (equivalent to 0.1 M in total volume of above mixture) was added, and the solution was further stirred at room temperature for 1 hour under continuous nitrogen purging. The mixture was subjected to centrifugation. The supernatant liquid was discarded, and the pellet was stored in an inert atmosphere (not dried). Anode paste was prepared using ~2 g of the washed iron pellet, 0.1 gm of powdered iron metal, and ~0.1 gm of KB or GUITAR-coated KB in 5 mL AIB 3.0 electrolyte solution at pH 7.5-8.0 containing 10 mM MV.

# 2.6. AIB 3.0 Cell

The cell containers were constructed from acrylic sheets [Housing] cut using a CO_2_ laser cutter (Boss Laser LS 1416, Sanford, FL). The battery cell assembly is described in a previous iteration and shown below in Section 3.^8^ The total volume of each half-cell was 1 mL [25mm × 25mm × 1.6 mm].

# 2.7. Membrane

Fumasep FAS-50 anion exchange membrane was cut into 27 mm × 27 mm pieces. This was submerged into AIB 3.0 electrolyte for 24 hours prior to use. The membrane was sandwiched between cathode and anode compartment.

**3. Cell Housing Assembly**

Cell housing preparation and final cell assembly is largely identical to supplemental build instructions in All Iron Battery 2.0 (with slight differences in cell size and volume)[1]. We have included section 3 and 4 in this supplemental information for the reader’s convenience with only minor changes.

The laser cut acrylic from Section 1 was assembled along with adhesive, current collector, and electrode pastes. Adhesive (clear seal sealant or other waterproof caulk) was applied in the interior side (Figure S.1 step 1). Then the current collector was applied to the adhesive (step 2). Further adhesive was then applied to the current collector to generate a seal to the second layer of acrylic (step 3). The graphite is thus sandwiched between the sheets of acrylic housing in contact with the glue (step 4). The acrylic-glue-graphite-glue-acrylic sandwich assembly was aligned and held together in position with binder clips for ~12 hrs to ensure that the glue was fully cured. Copper foil was added to the graphite to reinforce it for mechanical connection with alligator clips (step 5). Two such assemblies were constructed, one each for anode and cathode (steps 6-10).


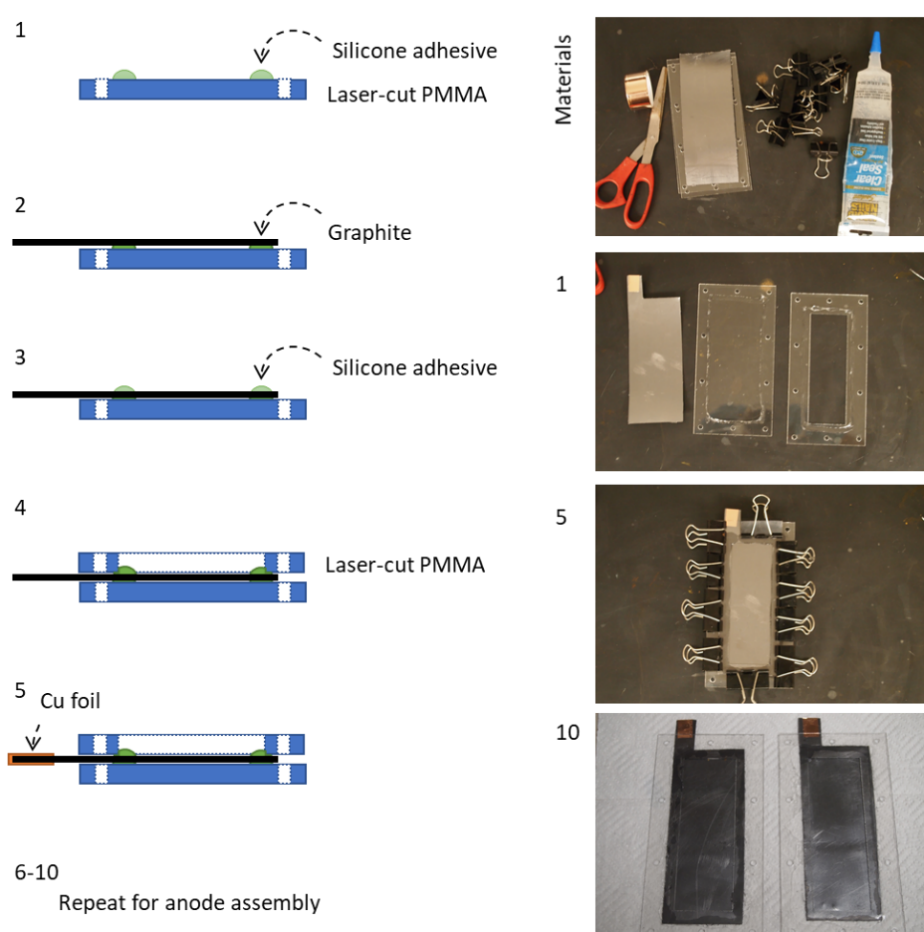


*Figure S.1: Cell housing assembly. Left shows schematic of the assembly process through the numbered steps. Images at right show images of the materials and selected steps of the process.*

**4. Battery Cell Final Assembly**

One finished half-cell housing was taken and labelled as cathode. ~1ml of catholyte paste was added and evenly distributed in the void space (Figure S.2 step 11).

Similarly, another half-cell housing was assembled as above. This should be labelled as anode. ~1 ml of anolyte paste was evenly distributed in the void space (step 12).

A small amount of silicone adhesive was applied on the inner boundary of the cathode cell (step 13) and a pre-cut 2.7 cm ×2.7 cm membrane was kept on the top of it (step 14).

Again, a small amount of silicone adhesive was applied on the boundary of the membrane (step 15) and the anode half-cell was carefully placed on the top of cathode half-cell with membrane (step 16) and held in place with binder clips. The assembly was fitted with screws.


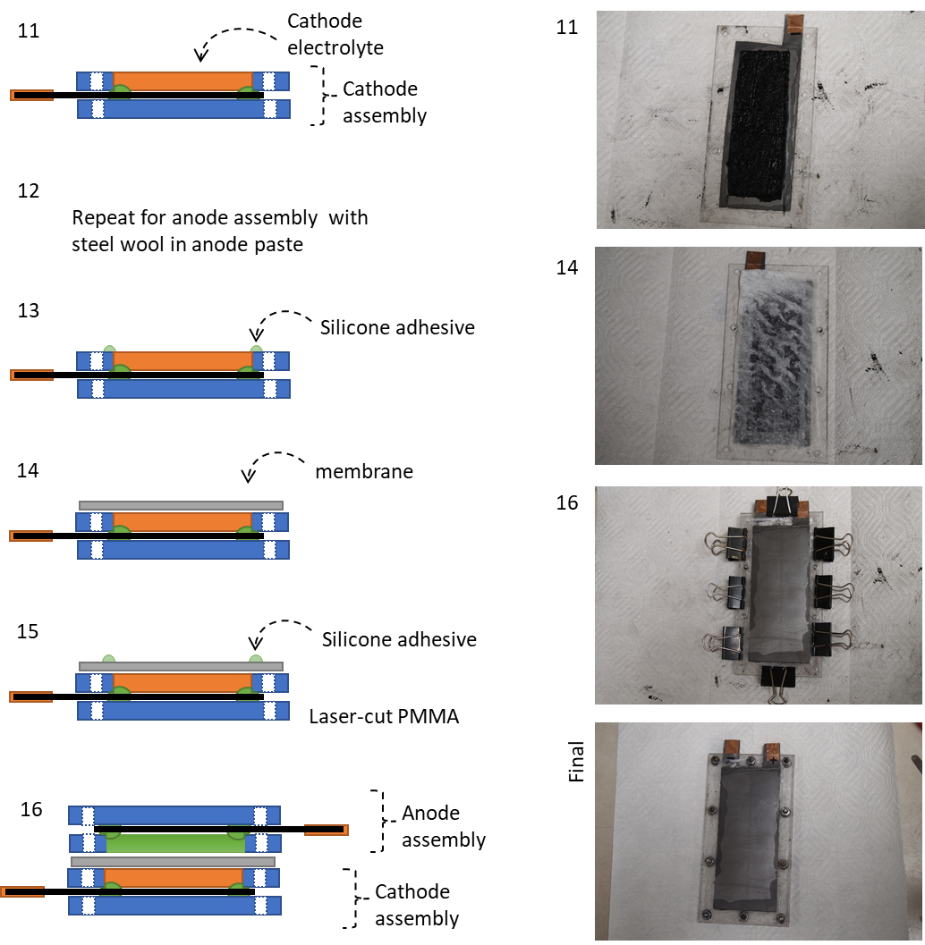


*Figure S.2: Assembly of Cell. Left shows schematic of the assembly process through the numbered steps. Images at right show images of the materials and selected steps of the process and the final, assembled cell.*

**5. Validation and Characterization**

- 1. **The precipitation routes of Iron chloride give chemical species with different electrochemical properties.**

Neutralization of FeCl_2_ with 15M KOH to make ~2M iron precipitate yields species that are relatively electrochemically inactive. The red curves in Figure S.3 below represent the FeCl_2_ neutralized with 15M KOH. In both the anode (left) and cathode (right) the red line shows sluggish kinetics and low overall current response.

When the order of operations was reversed and FeCl_2_ was added to 3M KOH solution in situ, the performance was improved. The FeCl_2_ was added until the pH was 7.5 with ~2M. The iron species thus evolved were separated and washed and transferred to the electrolyte solution. The material was then evaluated with equivalent CV settings, and this produced the green curves in Figure S.3. The kinetics and redox potentials were all improved.


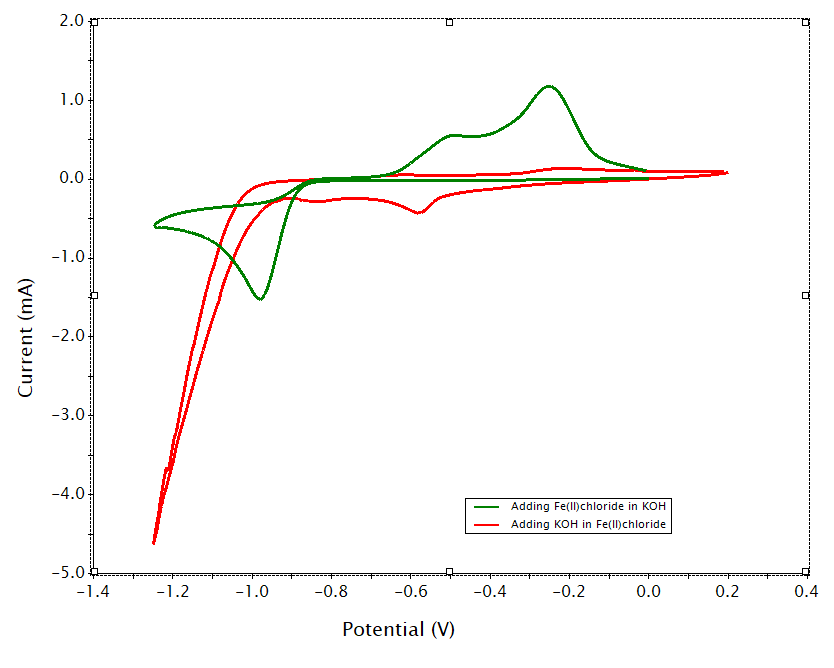

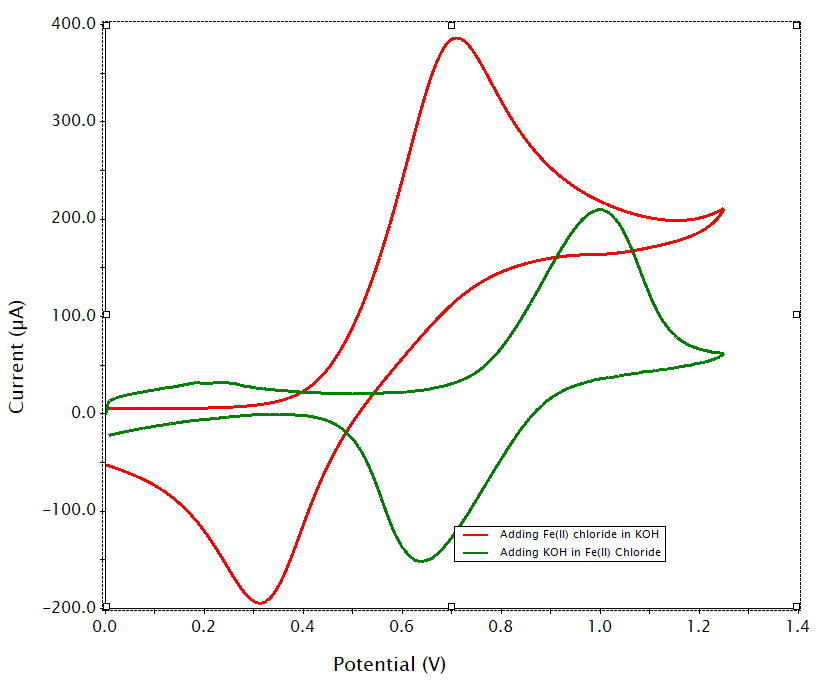


*Figure S.3: Cyclic voltammetry studies of iron (II) precipitate in 2M KCl solution containing 20% (v/v) ethylene glycol. (Left) is the anode paste; red is the CV result from the case where KOH was added to a beaker of FeCl_2_ solution; green is the CV result when FeCl_2_ solution was added to a beaker of KOH. (Right) is the equivalent case for the cathode paste. In both cases, the green curve indicates better performance.*

- 1. **Redox mediator improves the kinetics of the electroactive iron species.**

As the kinetics increase, the rate of electron transfer process at the electrode surface increases. This causes the peak separation (ΔEp) to decrease in Figure S.4.


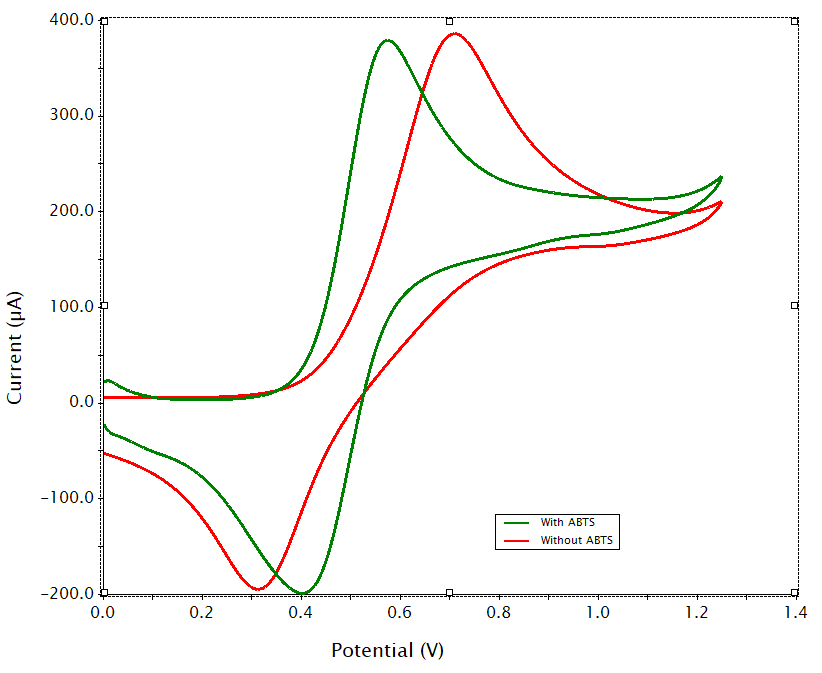

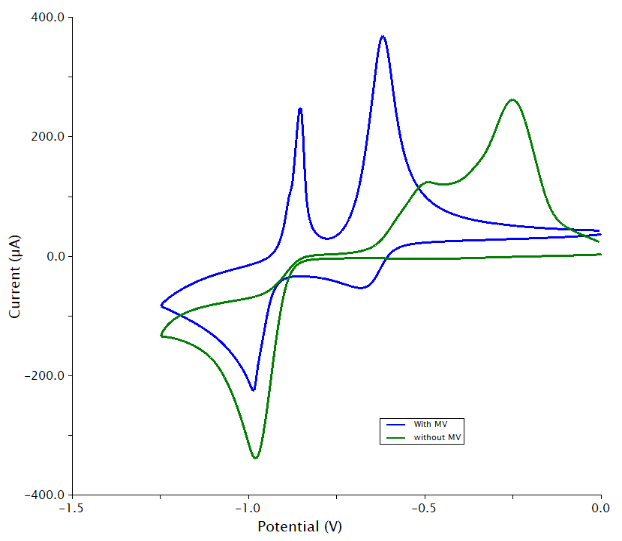


*Figure S.4: Addition of redox mediator lowers the ΔEp of the electrode active mediator, indicative of faster electro-kinetics.*

- 1. **The addition of GUITAR suppresses hydrogen evolution and improves electrode kinetics.**

GUITAR is known to have a larger potential window and is corrosion resistant[2]. The addition of GUITAR in the negative electrode of the cell increases the power density by ~2%.

- 1. **Addition of ethylene glycol improves conductivity and performance of iron precipitate in negative electrode.**

Cyclic voltammetry provides valuable information on electrochemical conductivity and performance of electroactive material. The higher peak current in Figure S.5 indicates the more facile electron transfer, which is the indication of improved conductivity.


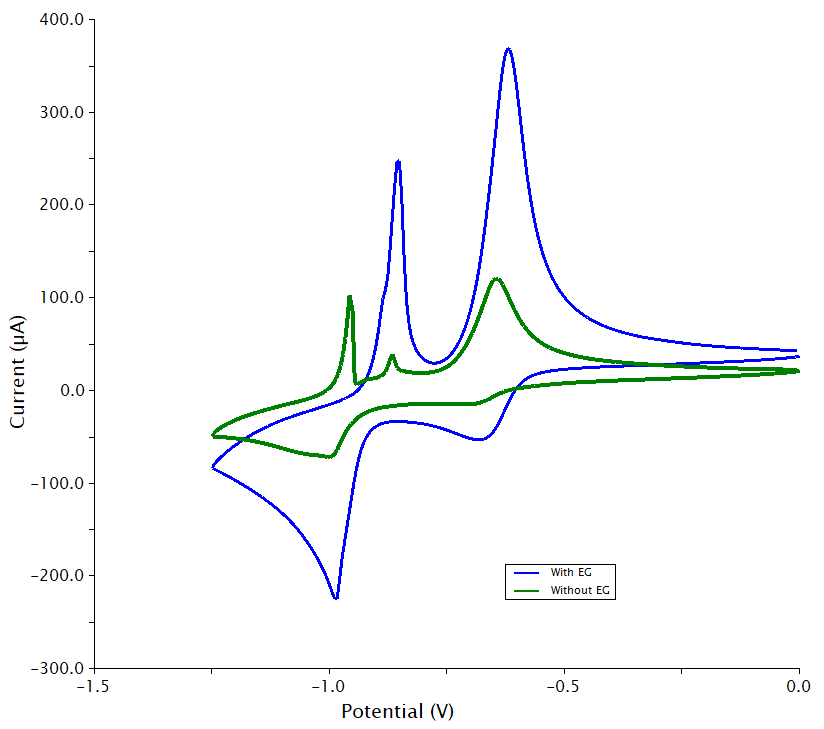


*Figure S.5: Addition of EG (blue CV trace) results in higher current than the equivalent material without EG (green CV trace) which yields better cell performance.*

**6. References**

[1] D. Koirala, N. Yensen, P.B. Allen, Open source all-iron battery 2.0, HardwareX 9 (2021) e00171. https://doi.org/10.1016/j.ohx.2020.e00171.

[2] H. Kabir, H. Zhu, J. May, K. Hamal, Y. Kan, T. Williams, E. Echeverria, D.N. McIlroy, D. Estrada, P.H. Davis, T. Pandhi, K. Yocham, K. Higginbotham, A. Clearfield, I.F. Cheng, The sp2-sp3 carbon hybridization content of nanocrystalline graphite from pyrolyzed vegetable oil, comparison of electrochemistry and physical properties with other carbon forms and allotropes, Carbon 144 (2019) 831–840. https://doi.org/10.1016/j.carbon.2018.12.058.
